# Supplementary material for: Smooth Interpolating Curves with Local Control and Monotone Alternating Curvature
Source: Comput Graph Forum. 2022 Oct 6;41(5):25–38. doi: 10.1111/cgf.14600 (PMC9827861; doi:10.1111/cgf.14600)
Supplement: Supplementary file 1 — Supplement Material [file CGF-41-25-s001.zip › Local-Smooth-Interpolating-MonoCurvature/extern/clothoids/docs/api-matlab/class_a00076.html]

Class CircleArc — Clothoids v2.0.9

### Navigation

- index
- toc
- next
- previous
- Clothoids »
- MATLAB API »
- Class CircleArc

# Class CircleArc¶

- Defined in File CircleArc.m

## Inheritance Relationships¶

### Base Type¶

- `public CurveBase` (Class CurveBase)

## Class Documentation¶

class CircleArc : public CurveBase¶
:   Public Functions

    function CircleArc( varargin)¶
    :   Create a new C++ class instance for the circle arc object

        **Usage:**

        ```
        ref = CircleArc() % create empty circle
        ref = CircleArc( x0, y0, theta0, k0, L ) % circle passing from (x0,y0) 
                                                 % at angle theta0 with curvature k0
                                                 % and length L
        ```

        **On input:**

        - `x0`, `y0`: coordinate of initial point
        - `theta0`: orientation of the circle at initial point
        - `k0`: curvature of the circle at initial point
        - `L`: length of curve from initial to final point

        **On output:**

        - ref: reference handle to the object instance

    function is\_type( ignoredArg)¶

    function build( self, x0, y0, theta0, k0, L)¶
    :   Build the circle from known parameters

        **Usage:**

        ```
        ref.build( x0, y0, theta0, k0, L )
        ```

        build a circle passing from (x0,y0) at angle theta0 with curvature and length

        **On input:**

        - `x0`, `y0`: coordinate of initial point
        - `theta0`: orientation of the circle at initial point
        - `k0`: curvature of the circle at initial point
        - `L`: length of curve from initial to final point

    function build\_G1( self, varargin)¶
    :   Build the circle from known parameters

        **Usage:**

        ```
        ref.build( x0, y0, theta0, x1, y1 ); % circle passing to [x0,y0] and [x1,y1]
                                             % with angle theta0 at [x0,y0]
        ref.build( p0, theta0, p1 ); % circle passing to p0 and p1 with angle theta0 at p0
        ```

        **On input:**

        - `x0`, `y0`: coordinate of initial point
        - `theta0`: orientation of the circle at initial point
        - `k0`: curvature of the circle at initial point
        - `L`: length of curve from initial to final point
        - `p0`: 2D point
        - `p1`: 2D point

    function build\_3P( self, varargin)¶
    :   Build the circle arc given 3 points. The point can be alingned in this case a degenerate straight arc if build.

        **Usage:**

        ```
        ref.build_3P( x0, y0, x1, y1, x2, y2 )
        ref.build_3P( p0, p1, p2 )
        ```

    function scale( self, sc)¶
    :   Scale circle by `sc` factor

        **Usage:**

        ```
        ref.scale( sc );
        ```

    function changeCurvilinearOrigin( self, s0, L)¶
    :   Change the origin of the circle curve to `s0` and set arc lenght to `L`

        **Usage:**

        ```
        ref.changeCurvilinearOrigin( s0, L );
        ```

    function to\_nurbs( self)¶
    :   return a nurbs representation of the circle arc

    function plot( self, npts, fmt)¶
    :   Plot the arc

        **Usage:**

        ```
        ref.plot( npts );

        fmt = {'Color','blue','Linewidth',2};
        ref.plot( npts, fmt );
        ```

        - `npts`: number of sampling points for plotting
        - `fmt` : format of the arc

    function plotPolygon( self, varargin)¶
    :   Plot the polygon of the NURBS for the arc

        **Usage:**

        ```
        ref.plotPolygon();
        ref.plotPolygon( 'Color','blue','Linewidth',2 );
        ```

        - `fmt` : format of the arc

    function obj\_handle( self)¶
    :   Return the `pointer` of the interbal stored c++ object

        **Usage**

        ```
        obj = ref.obj_handle();
        ```

    function copy( self, C)¶
    :   Make of copy of a curve object

        **Usage**

        ```
        ref.copy( C );
        ```

        where `C` id the curve object to be copied.

    function bbox( self, varargin)¶
    :   Return the bounding box of the curve object

        **Usage**

        ```
        [ xmin, ymin, xmax, ymax ] = ref.bbox( C );
        [ xmin, ymin, xmax, ymax ] = ref.bbox( C, offs );
        [ xmin, ymin, xmax, ymax ] = ref.bbox( C, offs, 'ISO' );
        [ xmin, ymin, xmax, ymax ] = ref.bbox( C, offs, 'SAE' );
        ```

        - xmin: x minimum coordinate of the bounding box
        - ymin: y minimum coordinate of the bounding box
        - xmax: x maximum coordinate of the bounding box
        - ymax: y maximum coordinate of the bounding box

        **Optional Arguments**

        - offs: offset of the curve used in the bbox computation
        - ’ISO’/’SAE’: use ISO or SAE orientation of the normal for offset computation

    function translate( self, tx, ty)¶
    :   Translate the curve by `(tx,ty)`

        **Usage**

        ```
        ref.translate( tx, ty );
        ```

    function trim( self, smin, smax)¶
    :   Cut the curve at the curvilinear parameter `smin` up to `smax`

        **Usage**

        ref.trim( smin, smax );

    function rotate( self, angle, cx, cy)¶
    :   Rotate the curve by angle `angle` around point `(cx, cy)`

        **Usage**

        ```
        ref.rotate( angle, cx, cy );
        ```

    function reverse( self)¶
    :   Reverse the direction of travel of the curve.

        **Usage**

        ```
        ref.reverse();
        ```

    function changeOrigin( self, newX0, newY0)¶
    :   Translate the curve in such a way the origin is at `(newX0,newY0`.

        **Usage**

        ```
        ref.changeOrigin( newX0, newY0 );
        ```

    function evaluate( self, s, varargin)¶
    :   Evaluate the curve at curvilinear coordinate `s`. Argument `s` may be a vector for multiple evaluations.

        **Usage**

        ```
        [ x, y, theta, kappa ] = ref.evaluate( s );
        [ x, y, theta, kappa ] = ref.evaluate( s, offs );
        [ x, y, theta, kappa ] = ref.evaluate( s, offs, 'ISO' );
        [ x, y, theta, kappa ] = ref.evaluate( s, offs, 'SAE' );
        ```

        **Optional Arguments**

        - offs: offset of the curve used in computation
        - ’ISO’/’SAE’: use ISO or SAE orientation of the normal for offset computation

    function eval( self, varargin)¶
    :   Evaluate the curve at curvilinear coordinate `s`. Argument `s` may be a vector for multiple evaluations.

        **Usage**

        ```
        XY = ref.eval( s );
        XY = ref.eval( s, offs );
        XY = ref.eval( s, offs, 'ISO' );
        XY = ref.eval( s, offs, 'SAE' );

        [X,Y] = ref.eval( s );
        [X,Y] = ref.eval( s, offs );
        [X,Y] = ref.eval( s, offs, 'ISO' );
        [X,Y] = ref.eval( s, offs, 'SAE' );
        ```

        **Optional Arguments**

        - offs: offset of the curve used compiutation
        - ’ISO’/’SAE’: use ISO or SAE orientation of the normal for offset computation

        **Output**

        - XY: matrix `2 x n` of the evaluated points
        - X: vector of the x-coordinates of the evaluated points
        - Y: vector of the y-coordinates of the evaluated points

    function eval\_D( self, varargin)¶
    :   Evaluate the first derivatives of the curve at curvilinear coordinate `s`. Argument `s` may be a vector for multiple evaluations.

        **Usage**

        ```
        XY = ref.eval_D( s );
        XY = ref.eval_D( s, offs );
        XY = ref.eval_D( s, offs, 'ISO' );
        XY = ref.eval_D( s, offs, 'SAE' );

        [X,Y] = ref.eval_D( s );
        [X,Y] = ref.eval_D( s, offs );
        [X,Y] = ref.eval_D( s, offs, 'ISO' );
        [X,Y] = ref.eval_D( s, offs, 'SAE' );
        ```

        **Optional Arguments**

        - `offs`: offset of the curve used in computation
        - ’ISO’/’SAE’: use ISO or SAE orientation of the normal for offset computation

        **Output**

        - `XY`: matrix `2 x n` of the evaluated points
        - `X`: vector of the x-coordinates of the evaluated point derivatives
        - `Y`: vector of the y-coordinates of the evaluated point derivatives

    function eval\_DD( self, varargin)¶
    :   Evaluate the second derivatives of the curve at curvilinear coordinate `s`. Argument `s` may be a vector for multiple evaluations.

        **Usage**

        ```
        XY = ref.eval_DD( s );
        XY = ref.eval_DD( s, offs );
        XY = ref.eval_DD( s, offs, 'ISO' );
        XY = ref.eval_DD( s, offs, 'SAE' );

        [X,Y] = ref.eval_DD( s );
        [X,Y] = ref.eval_DD( s, offs );
        [X,Y] = ref.eval_DD( s, offs, 'ISO' );
        [X,Y] = ref.eval_DD( s, offs, 'SAE' );
        ```

        **Optional Arguments**

        - `offs`: offset of the curve used in computation
        - ’ISO’/’SAE’: use ISO or SAE orientation of the normal for offset computation

        **Output**

        - `XY`: matrix `2 x n` of the evaluated points
        - `X`: vector of the x-coordinates of the evaluated point derivatives
        - `Y`: vector of the y-coordinates of the evaluated point derivatives

    function eval\_DDD( self, varargin)¶
    :   Evaluate the third derivatives of the curve at curvilinear coordinate `s`. Argument `s` may be a vector for multiple evaluations.

        **Usage**

        ```
        XY = ref.eval_DDD( s );
        XY = ref.eval_DDD( s, offs );
        XY = ref.eval_DDD( s, offs, 'ISO' );
        XY = ref.eval_DDD( s, offs, 'SAE' );

        [X,Y] = ref.eval_DDD( s );
        [X,Y] = ref.eval_DDD( s, offs );
        [X,Y] = ref.eval_DDD( s, offs, 'ISO' );
        [X,Y] = ref.eval_DDD( s, offs, 'SAE' );
        ```

        **Optional Arguments**

        - `offs`: offset of the curve used in computation
        - ’ISO’/’SAE’: use ISO or SAE orientation of the normal for offset computation

        **Output**

        - `XY`: matrix `2 x n` of the evaluated points
        - `X`: vector of the x-coordinates of the evaluated point derivatives
        - `Y`: vector of the y-coordinates of the evaluated point derivatives

    function theta( self, s)¶
    :   Evaluate the angle of the curve at curvilinear coordinate `s`. Argument `s` may be a vector for multiple evaluations.

        **Usage**

        ```
        theta = ref.theta( s );
        ```

    function theta\_D( self, s)¶
    :   Evaluate the angle derivatives (curvature) of the curve at curvilinear coordinate `s`. Argument `s` may be a vector for multiple evaluations.

        **Usage**

        ```
        theta = ref.theta_D( s );
        ```

    function theta\_DD( self, s)¶
    :   Evaluate the angle second derivatuve of the curve at curvilinear coordinate `s`. Argument `s` may be a vector for multiple evaluations.

        **Usage**

        ```
        theta = ref.theta_DD( s );
        ```

    function theta\_DDD( self, s)¶
    :   Evaluate the angle third derivative of the curve at curvilinear coordinate `s`. Argument `s` may be a vector for multiple evaluations.

        **Usage**

        ```
        theta = ref.theta_DDD( s );
        ```

    function kappa( self, s)¶
    :   Evaluate the curvature of the curve at curvilinear coordinate `s`. Argument `s` may be a vector for multiple evaluations.

        **Usage**

        ```
        theta = ref.kappa( s );
        ```

    function kappa\_D( self, s)¶
    :   Evaluate the curvature derivative of the curve at curvilinear coordinate `s`. Argument `s` may be a vector for multiple evaluations.

        **Usage**

        ```
        theta = ref.kappa_D( s );
        ```

    function kappa\_DD( self, s)¶
    :   Evaluate the curvature second derivative of the curve at curvilinear coordinate `s`. Argument `s` may be a vector for multiple evaluations.

        **Usage**

        ```
        theta = ref.kappa_DD( s );
        ```

    function xyBegin( self)¶
    :   Evaluate initial point of the curve.

        **Usage**

        ```
        [ x0, y0 ] = ref.xyBegin();
        ```

    function xyEnd( self)¶
    :   Evaluate final point of the curve.

        **Usage**

        ```
        [ x1, y1 ] = ref.xyEnd();
        ```

    function xBegin( self)¶
    :   Evaluate initial x-coordinate of the curve.

        **Usage**

        ```
        x0 = ref.xBegin();
        ```

    function xEnd( self)¶
    :   Evaluate final x-coordinate of the curve.

        **Usage**

        ```
        x1 = ref.xEnd();
        ```

    function yBegin( self)¶
    :   Evaluate initial y-coordinate of the curve.

        **Usage**

        ```
        y0 = ref.yBegin();
        ```

    function yEnd( self)¶
    :   Evaluate final y-coordinate of the curve.

        **Usage**

        ```
        y1 = ref.yEnd();
        ```

    function thetaBegin( self)¶
    :   Evaluate initial angle of the curve.

        **Usage**

        ```
        theta = ref.thetaBegin();
        ```

    function thetaEnd( self)¶
    :   Evaluate final angle of the curve.

        **Usage**

        ```
        theta = ref.thetaEnd();
        ```

    function kappaBegin( self)¶
    :   Evaluate initial curvature of the curve.

        **Usage**

        ```
        kappa0 = ref.kappaBegin();
        ```

    function kappaEnd( self)¶
    :   Evaluate final curvature of the curve.

        **Usage**

        ```
        kappa1 = ref.kappaEnd();
        ```

    function length( self, varargin)¶
    :   Return the length of the curve.

        **Usage**

        ```
        length = ref.length();
        ```

    function points( self)¶

    function bbTriangles( self, varargin)¶
    :   Evaluate the bounding box triangles of curve.

        **Usage**

        ```
        [P1,P2,P3] = ref.bbTriangles();
        [P1,P2,P3] = ref.bbTriangles(max_angle,max_size);
        [P1,P2,P3] = ref.bbTriangles(max_angle,max_size,offs);
        [P1,P2,P3] = ref.bbTriangles(max_angle,max_size,offs,'ISO');
        [P1,P2,P3] = ref.bbTriangles(max_angle,max_size,offs,'SAE');
        ```

        **Optional Arguments**

        - `max_angle`: maximum curve angle variation admitted in a triangle
        - `max_size`: maximum triangles size
        - `offs`: offset of the curve used in computation
        - ’ISO’/’SAE’: use ISO or SAE orientation of the normal for the offset

        **Output**

        - `P1`: `2 x n` matrix with the first points of the triangles
        - `P2`: `2 x n` matrix with the second points of the triangles
        - `P3`: `2 x n` matrix with the third points of the triangles

    function closestPoint( self, qx, qy, varargin)¶
    :   Evaluate the point at minimum distance of another point on the curve. `qx` and `qy` may be vectors so that the return values are vectors too.

        **Usage**

        ```
        [ x, y, s, t, iflag, dst ] = ref.closestPoint( qx, qy );
        [ x, y, s, t, iflag, dst ] = ref.closestPoint( qx, qy, offs );
        [ x, y, s, t, iflag, dst ] = ref.closestPoint( qx, qy, offs, 'ISO' );
        [ x, y, s, t, iflag, dst ] = ref.closestPoint( qx, qy, offs, 'SAE' );
        ```

        **Optional Arguments**

        - offs: offset of the curve used in computation
        - ’ISO’/’SAE’: use ISO or SAE orientation of the normal for the offset

        **Output**

        - `x`, `y`: Point at minimum distance from `(qx,qy)` on the curve.
        - `s`, `t`: Curvilinear coordinates of the point `(qx,qy)`.
        - `iflag`: `iflag < 0` some error in computation, iflag >0 is the numer of segment containing the point at minimum distance.
        - `dst`: point curve distance.

    function distance( self, qx, qy, varargin)¶
    :   Evaluate the distance of a point `(qx,qy)` to the curve. `qx` and `qy` may be vectors so that the return values are vectors too.

        **Usage**

        ```
        dst = ref.distance( qx, qy );
        dst = ref.distance( qx, qy, offs );
        dst = ref.distance( qx, qy, offs, 'ISO' );
        dst = ref.distance( qx, qy, offs, 'SAE' );
        ```

        **Optional Arguments**

        - `offs`: offset of the curve used in computation
        - ’ISO’/’SAE’: use ISO or SAE orientation of the normal for the offset

    function collision( self, OBJ, varargin)¶
    :   Check if two curve collide.

        **Usage**

        ```
        ok = ref.collision( obj );
        ok = ref.collision( obj, offs, offs1 );
        ok = ref.collision( obj, offs, offs1, 'ISO' );
        ok = ref.collision( obj, offs, offs1, 'SAE' );
        ```

        **Optional Arguments**

        - `offs`, `offs1`: offset of the curves used in computation
        - ’ISO’/’SAE’: use ISO or SAE orientation of the normal for the offsets

    function intersect( self, OBJ, varargin)¶
    :   Intersect two curves.

        **Usage**

        ```
        [s1,s2] = ref.intersect( obj );
        [s1,s2] = ref.intersect( obj, offs, offs1 );
        [s1,s2] = ref.intersect( obj, offs, offs1, 'ISO' );
        [s1,s2] = ref.intersect( obj, offs, offs1, 'SAE' );
        ```

        - `s1`: curvilinear coordinates of the intersections on the first curve
        - `s2`: curvilinear coordinates of the intersections on the second curve

        **Optional Argument**

        - `offs`, `offs1`: offset of the curves used in computation
        - ’ISO’/’SAE’: use ISO or SAE orientation of the normal for the offsets

    function info( self)¶
    :   Print on the console some information on the stored curve.

        **Usage**

        ```
        ref.info();
        ```

    function find\_coord( self, x, y)¶
    :   Get the curvilinear coordinates of the point `(x,y)`

        **Usage:**

        ```
        [s,t] = ref.find_coord( x, y );
        ```

        - `s`: curvilinear coordinate along the curve
        - `t`: curvilinear coordinate along the normal of the curve

    function yesAABBtree( self)¶
    :   Activate the use of AABB three in intersection/collision computations

    function noAABBtree( self)¶
    :   Deactivate the use of AABB three in intersection/collision computations

    function plotTBox( self, P1, P2, P3, varargin)¶
    :   Plot a triangle BBOX

        **Usage:**

        ```
        ref.plotTBox( P1, P2, P3 );
        ref.plotTBox( P1, P2, P3, 'Color', 'red' );
        ```

    function plotBBox( self, varargin)¶
    :   Plot the bounding box of the curve

        **Usage:**

        ```
        ref.plotBBox();
        ref.plotBBox('Color', 'red' );
        ```

    function plotTriangles( self, varargin)¶
    :   Plot the covering triangles of the curve

        **Usage:**

        ```
        ref.plotTriangles()
        ref.plotTriangles('red','FaceAlpha', 0.5);
        ```

### Quick search

### Table of Contents

- Matlab Interface Manual
- C++ API
- MATLAB API

«
hide menu

menu
sidebar
»

### Navigation

- index
- toc
- next
- previous
- Clothoids »
- MATLAB API »
- Class CircleArc

© Copyright 2021, Enrico Bertolazzi and Marco Frego.
Created using Sphinx 4.2.0.
